# Facilitators and Barriers to Implementing a Community Suicide Database and Prevention Program in Diverse Tribal Communities

**DOI:** 10.3390/ijerph21121616

**Published:** 2024-12-03

**Authors:** Meredith Stifter, Novalene Goklish, Charity Watchman, Kristin Mitchell, Jennifer Duncan, Michelle Miller, Mary HorseChief, Christopher G. Kemp, Mary Cwik, Emily E. Haroz

**Affiliations:** 1 Johns Hopkins Center for Indigenous Health, 415 N. Washington St., Baltimore, MD 21231, USAmcwik1@jhu.edu (M.C.); eharoz1@jhu.edu (E.E.H.); 2 White Mountain Apache Center for Indigenous Health, Whiteriver, AZ 85941, USA; 3 Navajo Nation, Shiprock Center for Indigenous Health, Shiprock, NM 87420, USA; 4 Navajo Nation, Chinle Center for Indigenous Health, Chinle, AZ 86503, USA; 5 San Carlos Apache Tribe, Life Is Precious, 203 Medicine Way Rd., Peridot, AZ 85542, USA; 6 Hualapai Tribe, Behavioral Health, 488 Hualapai Way, Peach Springs, AZ 86434, USA; 7 Cherokee Nation, Tahlequah, OK 74464, USA

**Keywords:** American Indian, suicide prevention, implementation science, mental health, suicidal behaviors, native American

## Abstract

Suicide is the second leading cause of death for American Indian youth, far surpassing the rates of suicide experienced by other races. The White Mountain Apache Tribe has made significant impacts on suicide risk by implementing a robust suicide prevention program which includes a community-led database and case management follow-ups. Due to the success of the program in preventing suicides, the White Mountain Apache team has worked with other tribal communities to adapt the program. We wanted to understand the factors that are most important to implementing and sustaining this model and how these factors compare with existing implementation science frameworks. We employed an adapted nominal group technique to compile facilitators and barriers to implementation of the suicide prevention model across settings with five partner teams. Two researchers independently coded the resulting list of facilitators and barriers using the Consolidated Framework for Implementation Research (version 1.0) codebook. The final list of cross-site prioritized facilitators and barriers included 41 factors. Some factors did not match easily with the framework’s constructs. The White Mountain Apache suicide prevention team noted that seven of the top prioritized factors are considerations they most try to emphasize to new communities working in suicide prevention. The factors fall into two key themes: staffing and tribal engagement. This finding affirms their focus when they conduct suicide prevention trainings with new communities and provides an opportunity for more structure and in-depth training in those two areas. Several factors could not be easily coded to the framework, especially around the sociocultural characteristics of suicide prevention work in Native communities. This contributes to the larger discussion in implementation science concerning the ways in which Indigenous approaches to public health differ from Western models.

## 1. Introduction

Suicide is the second leading cause of death for American Indian (AI) youth aged 10–24, with AI youth experiencing four times the rate of suicide compared to their peers of other races [1]. This pattern has been exacerbated by the COVID-19 pandemic. Trends from 2020 to 2022 show that suicide deaths in AI youth and young adults increased sharply and much more than for other races [2]. Suicide rates can vary widely across and within AI tribes, with some tribes experiencing higher rates than others, and some age groups or genders more affected than others. Given the complex nature of this problem, it is imperative that community-based suicide prevention efforts be based on local data that explain the nuances of the problem in this specific population. Suicide surveillance that includes systematic data collection, analysis, and dissemination is a powerful tool that allows stakeholders to understand the scope of suicide and can include identification of and connections to care for those at risk. The National Strategy for Suicide Prevention stresses the importance of improving the timeliness and usefulness of suicide surveillance data to maximize its potential to inform action at national and local levels [3].

The White Mountain Apache Tribe (WMAT) in Arizona has made significant impacts on suicide risk in their community by implementing a robust suicide prevention program which includes a community-led database. Between 2001 and 2006, they documented suicide deaths at more than twice the rate of all AI/Alaska Native (AN) tribes, and more than three times the rate of the US white population [4]. Those at highest risk of suicide were youth under 20 years old, compared with all US races where suicide rates have tended to be higher in older ages (45–64 for women and 75+ for men) [5]. The WMA Tribal Council responded to this spike in youth suicide by establishing the Celebrating Life suicide prevention program and case management follow-up team in partnership with Johns Hopkins University Center for Indigenous Health (CIH, formerly the Center for American Indian Health) [4]. This program has allowed the tribe to better understand what contributes to and prevents suicide on the reservation and has contributed to decreases in suicide attempts and deaths in the tribe [4].

Based on the success of the Celebrating Life suicide prevention program, the Center for Indigenous Health (CIH) and longstanding research partners, WMAT and Navajo Nation, received a five-year NIH grant to adapt Celebrating Life for Navajo Nation and build capacity in three other tribal communities to develop locally run suicide databases tailored to their unique community contexts, strengths, and resources (the Southwest Hub for American Indian Youth Suicide Prevention Research; U19MH113136). The three additional partners identified to participate in this project were the San Carlos Apache Tribe, the Hualapai Tribe, and Cherokee Nation. In addition, the Chinle Comprehensive Healthcare Facility, an Indian Health Service (IHS) hospital on Navajo Nation, received a SAMHSA Zero Suicide Initiative grant, part of which included collaborating with CIH on the suicide database and case management [6]. Compared with WMAT’s program, which has been operating for over 20 years, these partner sites have implemented their suicide prevention databases and case management much more recently (see Supplementary Figure S1 for more information on the implementation process). Because suicide has continued to represent a health disparity in Native communities, and Celebrating Life is a nationally recognized and award-winning program, over 30 tribal communities have expressed interest in adapting Celebrating Life in recent years.

Along with this growth, the project team wants to understand what factors and strategies have been instrumental in the program’s implementation and sustainability (K01MH116335). In this paper, we aimed to identify common barriers and facilitators of implementation across diverse settings. To consolidate our findings and tie them into the literature, we applied the Consolidated Framework for Implementation Research (CFIR) as we coded our findings [7,8]. Findings from this study can inform the implementation and sustainability of the Celebrating Life program as it continues to be disseminated. More broadly, the alignment of our findings with CFIR can also help us better understand the applicability of CFIR in Indigenous communities and contexts.

## 2. Materials and Methods

### 2.1. Overview

We employed an adapted nominal group technique to compile common and impactful facilitators and barriers to implementation and sustainability of the Celebrating Life model across partner settings. We then mapped these factors onto CFIR (version 1.0) to understand which CFIR constructs were relevant, which are less so, and which of the teams’ priorities were not reflected in CFIR. This study was approved by the White Mountain Apache Tribal Council and Health Board through resolutions and by the Johns Hopkins Bloomberg School of Public Health IRB. Its ClinicalTrials.gov identifier is NCT03755206, and its unique protocol ID is IRB00008138.

### 2.2. Conceptual Framework

**Figure 1:**
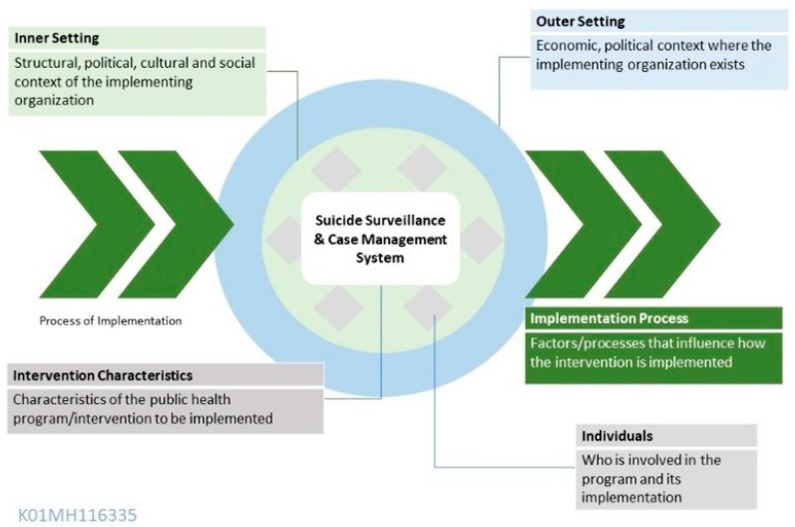
Consolidated Framework for Implementation Research model as applied to the Celebrating Life suicide prevention program. The five domains are listed and described and placed in the context of Celebrating Life as the program to be implemented.

The Consolidated Framework for Implementation Research is a determinant model specific to health services that organizes the constructs associated with effective implementation into five key domains. It was first published in 2009 as a guide for tailoring implementation strategies during adaptation, systematically assessing potential barriers and facilitators to implementation, and explaining outcomes of implementation projects [7]. The framework covers summative and formative outcomes and examines effectiveness in specific settings, sustainability, and dissemination to other settings (see Figure 1). The five domains are inner setting, outer setting, implementation process, individuals, and intervention (or innovation) characteristics. The CFIR guide recommends that researchers who engage with CFIR also critique and improve upon the framework, which has since led to the development of version 2.0 in 2022, with updated tools and explanatory content forthcoming [7,8]. While it is most often used in healthcare settings, it has other applications as well, including in low-income settings.

### 2.3. Partner Communities and Program Settings

#### 2.3.1. The WMAT Celebrating Life Program

The Fort Apache Indian Reservation sits east of Phoenix in Arizona, encompassing 2600 square miles, and is home to around 14,000 enrolled members [9]. With the 2001 creation of the suicide prevention task force (predecessor to the Celebrating Life program), the Tribal Council also passed a resolution mandating all first responders must report any suicide ideation, attempt, or death to the program. In 2003, the tribe transitioned to the Celebrating Life suicide prevention program; and in 2006, the resolution was expanded to include all community members as mandated reporters; for example, a friend sharing that they have suicidal thoughts. Individuals are referred to the prevention program, and through the resolution, Celebrating Life staff are mandated to refer those individuals to local behavioral health services and, if appropriate, alcohol/substance use treatment. A Celebrating Life case manager then follows up with the individual to confirm the details of the referral and helps with connection to these and any additional needed services. The assigned case manager adds individuals to their case load as soon as a referral comes in and can continue attempting to contact the at-risk individual for 90 days for those under 35 years old and 30 days for older adults. Case managers can also offer continued follow-up with the individual to check on their mental health and wellness after that initial visit [4].

#### 2.3.2. The Honoring Life Program in Shiprock, NM on the Navajo Nation

The Navajo Nation spans 27,000 square miles across Utah, Arizona, Nevada, and New Mexico. The total Shiprock, New Mexico, service area provides services to 31,000 people across 4200 square miles [10]. The Honoring Life program, which is the most similar to Celebrating Life of all the adapted programs, provides suicide prevention services across this Shiprock service area. As with Celebrating Life, team members of Honoring Life are CIH employees, so they benefit from the partnership history, infrastructure, and devoted staff to set up and implement the program. Honoring Life uses memorandums of understanding (MOUs) with tribal agencies, rather than a tribal mandate, to formalize their partnerships, primarily with the local IHS hospital, schools, and school districts. MOUs allow the team to receive referrals for youth and young adults ages 10–24 who have experienced recent suicide and self-harm behaviors and to provide case management services to those individuals (with parent permission as needed). With each referral, the team follows up (typically in person) with the individual to confirm the details of the report and understand the surrounding circumstances. The window for locating individuals is 90 days. If the individual agrees to regular case management follow-ups, those check-ins (in-person and via text) can occur weekly or every other week and become less frequent with time as the participant’s risk and need for services decreases.

#### 2.3.3. The Embrace Life Program in Chinle, AZ on the Navajo Nation

Chinle, Arizona, is a 16 square mile community central in the Navajo Nation reservation that is home to 4000 people [11,12]. The community also contains the Chinle Comprehensive Health Care Facility (CCHCF), which serves 37,000 patients [11]. The Zero Suicide Initiative (ZSI) supports the Embrace Life program, in which case managers monitor CCHCF patients with suicide behaviors from urgent care, the emergency department, outpatient clinics, and the counseling department. Providers within these departments use screening questionnaires in patient visits to determine suicide risk. Case managers in the program then follow up depending on risk level to ensure complete wraparound services for those patients. Highest-risk cases are attempted to be reached within 24–48 h. The healthcare system connects patients presenting with suicidal ideation with necessary services, and ZSI case managers ensure those connections are made and no services are missed. Their primary focus is to connect with patients who need to be transferred to inpatient care facilities, those who miss follow-up appointments, and those who are discharged home from services, making sure nobody experiences gaps in care. Case managers may conduct home visits for those hard-to-reach high-risk patients to ensure hollow-up to care and resources.

#### 2.3.4. The Cherokee Nation Suicide Prevention Program

The reservation of the Cherokee Nation comprises eight full counties and parts of six additional counties in Oklahoma [13]. As many as 144,000 of the 400,000 registered tribal citizens live within the nearly 7000 square miles of reservation land [14]. Surveys conducted in 2018 led the tribal nation to invest in a suicide prevention program. They showed that only 32% of adults experiencing mental health symptoms in one of the counties was receiving services through state-funded facilities, and between 37% and 60% of Native students in all county schools reported feeling anxious, hopeless, or worthless, with 141 students reporting they might hurt someone or themselves [15]. The Cherokee Nation database and case management system operates through partnerships between the grassroots coalition Grand Nation, Cherokee Nation Behavioral Health (CNBH), law enforcement, and other community resources. Core team members at CNBH receive referrals and collect data on suicide and self-injury from schools, medical providers, and first responders, and a small team of case managers follows up with calls and home visits to provide case management to high-risk individuals. Because the team employs fewer case managers than some of the other partner sites, the case managers’ time is limited, and they can only offer follow-up visits with those at high risk. The team also identifies and trains community coalitions in data collection and case management to expand services across all Cherokee Nation.

#### 2.3.5. The Hualapai Suicide Prevention Program

The Hualapai Reservation spans 1500 square miles, bordering the Grand Canyon and Colorado River. As many as 1353 of the 2300 enrolled members live on this land, as well as 268 non-enrolled people [16]. The Hualapai Tribe’s prevention program is embedded in the Tribal Behavioral Health Program and was adopted into the Behavioral Health Policies and Procedures through a tribal resolution. Case managers provide “intensive outpatient” care, including a minimum of two client meetings per week for the first several weeks post-suicide behavior or event. They accept referrals from community members, schools, and all tribal departments. The program employs a licensed social worker who provides direct counseling services and two paraprofessionals who can provide case management and follow up on referrals. They follow up on all reports, refer individuals to necessary services, and develop a safety plan before offering continued case management and counseling. Case managers have been able to meet with students both at school and at home, and flag higher risk youth with more frequent self-harm events for additional care and outreach to families. They also host a variety of school, holiday, youth, and parent events as extra resources and opportunities to connect in the community.

#### 2.3.6. The San Carlos Apache Life Is Precious Program

The San Carlos Apache Reservation borders the southern end of the WMAT’s Fort Apache Reservation. San Carlos covers 2800 square miles and is home to around 10,000 enrolled members [9,17]. San Carlos Apache Tribe’s suicide prevention program is called “Life is Precious” and is a task force housed within the San Carlos Wellness Center. They accept referrals from hospitals and first responders, among other tribal departments, and use intake and follow-up reporting forms based on Celebrating Life. They provide wraparound services, ensuring all care providers on a patient’s team are coordinating on the best care strategies. This coordination happens during daily team huddles to discuss high-risk and regular patients. They also host suicide prevention programming in the community, including events that promote awareness and dispel stigma, and creative group activities that can deter people from suicide ideation and substance use. Some popular support group themes are art therapy for specific youth- and adult-age groups, skateboarding groups, and groups that walk newly cut paths in the grounds around the Wellness Center. These groups also help the team transition individuals, especially youth, from higher-risk follow-ups, off of their services, to mentoring and other activities. For example, the art therapy group is aimed at higher-risk individuals. The Life is Precious team responds to all crises, not just suicide-related behaviors, so they did not ultimately have time to participate in the NGT. It is still important that we describe their program here as their work has made an integral contribution to the Southwest Hub and the learnings shared across suicide prevention programs.

### 2.4. Nominal Group Technique Process

The nominal group technique (NGT) approach aims to gather prioritized implementation barriers and facilitators across tribal contexts. Specifically, initial in-person group meetings were held with WMAT, Navajo Nation Chinle site, and the Navajo Nation Shiprock site. A virtual meeting was held with representatives from the programs at Cherokee and Hualapai. All meetings were held with stakeholders where the program had been running for at least 1 year. Stakeholders were selected to participate if they were aware of the program, participated in provision of services, or were program administrators. The NGT process was selected to encourage participation from all group members and reduce the potential domination by any one member [18].

During the initial meetings, participants were asked to free list their response to two prompts: What are all the things that contribute to your program running well? What are the challenges in having your program run well? For each prompt, participants brainstormed lists on their own and wrote them in their own notebooks. After the end of the brainstorming, the group took turns sharing their thoughts, and a comprehensive list was built on a white board at the front of the room (or on a word document if held virtually). We went around the room ensuring everyone had a chance to share, adding only those factors that were listed which were not already included on the whiteboard/document. After this process was complete, we paused to discuss any clarity that was needed for the factors listed together. Finally, we asked participants to privately vote on their top five factors they thought most important for successful implementation (or challenges to implementation) and then rank each of these five factors as very important, moderately important, or not very important. Votes and importance ratings were recorded on participants’ own paper and then submitted to the facilitators at the conclusion of that topic. This process was then repeated with challenges.

At the end of each meeting, prioritized facilitators and barriers were tallied separately. Ranking of importance was conducted by counting the number of participants that included the factor in their top five rankings and then providing an average weight of the importance factor. For example, “Community Awareness” was listed in the top five facilitators across 54% (7/13) of group participants. Across these seven participants, the average importance rating was “Very Strong”. This process was conducted in each of the group meetings such that at the conclusion of the meetings, we had lists of prioritized barriers and facilitators that represented these factors across settings.

For the cross-site analysis, we compiled all priorities from the groups and noted the number of sites endorsing each priority (some factors were only mentioned by one site, some by all five sites). We then coded these factors based on the CFIR v1.0’s domains and constructs using the CFIR codebook [7]. Two researchers independently coded the list of prioritized facilitators and barriers. Coding discrepancies were discussed and agreed upon then blind coded by a third researcher to confirm the final decisions. If a CFIR domain or construct did not align, this was noted.

## 3. Results

The final list of cross-site prioritized facilitators and barriers included 41 factors (see Supplementary Table S1). Twenty-five of the factors were endorsed by more than one team (see Table 1). Nineteen of the factors were facilitators for programs, twelve were barriers or challenges to program success, and ten were discussed as facilitators in some programs and challenges in others. For example, three teams brought up “team size” as a key factor; some said they had a good size team, which facilitated their program functioning, while others said they did not have enough case managers to balance the work needs, which was challenging the program’s success. All barriers or facilitators mentioned aligned with CFIR domains; however, the context in which they were mentioned made it hard to fully align certain domains.

Two factors were endorsed by all five teams: “dedicated and motivated staff” was a strong facilitator and aligned with the CFIR domain “Characteristic of Individuals”; and “collaboration across diverse departments and agencies” was a facilitator/barrier which aligned with the “Outer Setting” of the CFIR. The top factors that were endorsed only as barriers (compared with facilitator/barrier) were each endorsed by three teams: “community resistance and stigma related to suicide”; “patient engagement and follow through with case management”; and “challenges locating and contacting clients”. These three factors are related to each other in many ways. “Community resistance” was coded as in the outer-setting domain and can influence individuals being less open to case management, resulting in “patient engagement” and “challenges locating clients”, which were both coded in the implementation process domain. Some factors did not match easily with CFIR constructs because the intervention is intrinsically linked to community context and is not quite captured in the first version of this framework. Examples of these mismatched factors include “community trust of the program” and “sensitivity to diverse sociocultural norms”.

## 4. Discussion

The teams involved in this study were all trained in suicide prevention by the WMAT Celebrating Life team. They use similar reporting forms based on the Celebrating Life model, and they all receive referrals from hospitals and tribal agencies, among other sources like schools and community members. They make referrals to treatment and counseling as needed, and they are all working on both the database and case management. They also all generally follow a stepped care model, matching higher-risk individuals with more frequent case manager follow-ups.

The teams also differ in how they have implemented Celebrating Life: WMAT has a tribal resolution requiring the reporting of self-harm behaviors by all community members; Hualapai requires reporting by all tribal agencies; and Cherokee Nation requires reporting by the healthcare system. These teams did not have tribal resolutions in place when the work started; over the course of their partnerships with WMAT and CIH, they have recognized the need for structural support from resolutions and have worked with tribal governance to implement them. San Carlos Apache has a crisis response team responding to all emergencies, not just suicide and self-harm, and the Navajo Nation Shiprock program employs agreements with hospitals and schools to receive their referrals. The Shiprock team also limits their referrals to youth under 25 years old, while the other programs work with clients of all ages. The programs use different data systems based on their resources and the systems used by other local agencies. For example, WMAT uses REDCap because of their close partnership with Johns Hopkins University, whereas Cherokee Nation uses the electronic health record system because of their close working relationship with, and reporting from, the hospital. These varied contexts, in addition to the nominal group technique utilized in this study, help us understand what an essential element of the suicide database and prevention program is, as well as what is able to be adapted to the community’s needs and priorities.

The factors that the teams identified as being most important to the work matched each of the five domains of the Consolidated Framework for Implementation Research. The 41 factors spanned ecological levels, underscoring the complexity of integrating suicide prevention models into a community. The Celebrating Life team noted that seven of the top prioritized facilitators and barriers to implementation are factors they most try to emphasize to new communities working in suicide prevention as well. The factors fall into two key themes: staffing and tribal engagement.

Factors related to staffing include “having a dedicated and motivated staff”, “turnaround between at-risk individual identification and case manager contact”, “having enough staff members to provide detailed case management”, and the “training of case managers”. The Celebrating Life team emphasizes the importance of considering staffing when they meet with communities who are new to the suicide prevention program because implementation works best when the team meeting with Celebrating Life staff is the same team that will be delivering case management. Case managers spend ample time role playing in the office before they go out into the community to shadow home visits with more seasoned case managers. Suicide and self-harm are incredibly sensitive topics and sometimes result in crisis situations, and case managers need to be confident in the questions they ask and knowledgeable on safety procedures from practicing roleplay scenarios before they are prepared to leave the office. Additionally, as more referrals come into the suicide prevention program, staffing needs to grow to meet that need.

Factors related to tribal engagement include “community resistance and stigma related to suicide”, “tribal and political leadership”, and, importantly, “tribal resolution or mandate”. The Celebrating Life team recognizes the ways in which this program is strengthened by its tribal mandate and has seen many instances when implementation in other communities has faltered without one. The partner teams on this project were able to show their communities and leadership the need for structured suicide prevention and were able to build support for mandates during implementation. Mandates can also help with staffing by making case manager positions more stable rather than renegotiating them with every grant or funding cycle. Because of the sensitive nature of suicide and the belief in many Indigenous cultures that discussing suicide invites it into the community, tribal leadership is sometimes unwilling at first to fully support suicide prevention programming [19]. However, strong grassroots leadership can help move the process forward, build community buy-in, and ultimately build support in tribal leadership. Suicide prevention research and programming occurred in the White Mountain Apache Tribe for at least ten years before the first resolution was passed by Tribal Council to provide more structure to Celebrating Life. Establishing these programs can be a long process, requiring communities to be dedicated to this work beyond reacting to the suicide clusters or crises they may be experiencing when they first contact the Celebrating Life team for help.

We also identified additional factors that could not be easily coded for CFIR constructs and domains, especially around sociocultural characteristics of suicide prevention work in Native communities. For example, researchers agreed on the wider domains for “family resistance”, “parental consent”, and “sensitivity to diverse sociocultural norms” but did not settle on more specific constructs. This was true for four other factors as well. Ours is not the only work that has noted the ways Indigenous approaches to public health differ from Western models. Rubin Means et al. evaluated the usefulness of CFIR in low- and middle-income countries—some of which have a strong presence of Indigenous populations whereas others that are dissimilar from Western cultures—and identified two constructs unanimously believed to be compatible outside high-income countries and two constructs believed to be incompatible [20]. They also proposed a new domain called “Characteristics of Systems” that would help fill the gaps in CFIR’s applicability, capturing the ways systems constructs “inherently interact with existing constructs across domains”. These findings reflect our discussion of the factors that fell outside of CFIR largely because they influenced multiple domains at once.

Brockie et al. utilized different adapted models in their implementation of suicide prevention programming in the Fort Peck Tribes in Montana and identified sustainability factors that overlapped with our findings, including the factors we found to be unique from CFIR [21]. They adapted the UCLA Asset Mapping guide, adding questions on cultural assets, the responses to which specified the agencies that should be coordinated with for the success of a suicide prevention program. They also adapted the PSAT sustainability framework into a qualitative interview guide and found themes that are consistent with our work, like the importance of tribal and political leadership and support, regular and sustainable funding, clear policies and procedures, coordination with other departments, and community trust in the program. Additionally, they observed opportunities to incorporate traditional ways of honoring people, “focused on culture and community”, to build organizational capacity and reward and thank staff [21] (p. 9).

Although focused more on health determinants than interventions, Nisbett et al. propose that inequity is a key factor missing from most frameworks [22]. Suicide is one of the most inequitable burdens on Indigenous communities, so this is an important consideration for why we observed some mismatches between our findings and CFIR [23]. The researchers developed the Nutrition Equity Framework by bringing together existing frameworks, including Indigenous food systems, and emphasizing the “interactions, rather than the individual components, [which] are most critical for understanding the structural inequities causing malnutrition” [22] (p. 3). Once again, it seems existing Western models in many ways fail to account for constructs that span domains, making it challenging to capture elements that impact the intervention, the implementation process, and sociopolitical contexts within and outside of the program.

## 5. Strengths and Limitations

One strength of this study is that the participating teams come from varied backgrounds and adapted Celebrating Life differently. They provided perspectives that are reflective of differing implementation processes, and the data are richer in their variety and potentially more widely applicable. This is important as we are expanding the implementation of Celebrating Life and working with additional communities. This study, in addition to the work with new communities, continues to inform the packaging of Celebrating Life for wider adaptation. With so much interest from Native communities, this packaging will facilitate effective and efficient implementation. Celebrating Life can be part of a growing body of research to adapt and implement suicide prevention work in AI/AN communities, including Caring Contacts, which promotes social connectedness through e-messaging [24,25], and PC CARES, which builds local capacity to shift from crisis intervention to prevention-focused work [26]. Even in a program like PC CARES, which was designed with and for Alaska Native communities, it still must undergo some adaptation process to best make it fit in new Alaska Native regions [26].

Finally, this study fits into the iterative and ongoing CFIR evolution to be better applied across public health settings. CFIR developers acknowledge the need for feedback from implementation scientists to improve the model, and it is currently undergoing updates to the second version [8]. While the second version is still being finalized, developers stated that they drew on information from literature reviews of papers citing CFIR and surveys of authors who used CFIR in their studies. This study adds to that conversation and can be useful in informing future iterations of the model.

The limitations of the study include the fact that we were unable to discuss facilitators and barriers with one of the implementing teams. The San Carlos Apache Tribe is an important partner in this suicide prevention work, but their 24/7 crisis response design meant that they were unable to join the focus group calls during which we gathered the study data. We are grateful that they supported this study by reviewing this publication and providing feedback. A second limitation is that the time between data collection and coding left some factors open for interpretation to the coders. When researchers reviewed the data for coding, they realized that the wording was somewhat unclear for a few prioritized factors, making them more challenging to code. By reviewing focus group notes and coding by three researchers total, we sought to avoid researcher bias in this process. Finally, some of the factors were ultimately categorized as both facilitators and barriers. When some teams benefitted from a factor going well, and others were challenged by a factor not going well (i.e., facilitated by a tribal mandate vs. challenged by not having a tribal mandate), we listed the factor as a “facilitator/barrier”. We envisioned factors being clearly defined one way or the other, but in this categorization, we are able to keep the consideration high on the list when prioritized by multiple teams. In this way, we can identify it as a key consideration for implementation.

## 6. Conclusions

Based on the success of WMAT’s Celebrating Life suicide prevention program and the implementation efforts of other tribal communities, WMAT and four other teams adapting Celebrating Life shared the factors that have been key to the success or challenges of their tribally run suicide database and prevention programs. Understanding what these factors are and how they compare with existing implementation models is important for informing best practices in the future adaptation of Celebrating Life in other interested communities. We wanted to see which elements have been important when implementing Celebrating Life across different contexts.

Our findings revealed implementation factors that span ecological levels, as well as several unique constructs that are important to suicide prevention work in Native communities. Considerations around staffing and tribal engagement appear repeatedly in the top implementation factors identified by the participating communities and are affirmed by the Celebrating Life team as themes they most try to emphasize when training new communities in suicide prevention programming. The teams identified additional factors for suicide prevention implementation in Native communities that are not currently reflected in the CFIR framework, especially around sociocultural contexts. The CFIR developers acknowledge the importance of incorporating feedback into the framework to continually evolve it to be better applied, and this research aligns with that conversation. Inequities in suicide rates continue to widen; rates increased the most and remained the highest in AI/AN populations in 2021, while US white rates decreased [2]. Suicide prevention will continue to be a priority in Native communities, and we need useful implementation models to guide prevention work across the US.

## Figures and Tables

**Table 1:** Top prioritized facilitators and barriers to implementing Celebrating Life.

Prioritized Factor	Number of Sites Endorsing	Barrier or Facilitator	CFIR Domain
Dedicated and motivated staff	5	Facilitator	IV. Characteristics of Individuals
Coordination and regular meetings with other departments (MOUs and protocols); collaboration across diverse stakeholders	5	Facilitator/Barrier	II. Outer Setting
Availability and responsiveness of case managers and turnaround between identification and contact	4	Facilitator	V. Process
Community awareness of program	4	Facilitator/Barrier	II. Outer Setting
Enough staff members to provide detailed case management and balance different work needs	3	Facilitator/Barrier	III. Inner Setting
Community trust of the program	3	Facilitator	II. Outer Setting
Clear policies and procedures for a variety of situations	3	Facilitator	III. Inner Setting
Training of case managers	3	Facilitator	III. Inner Setting
Community resistance and stigma related to suicide	3	Barrier	II. Outer Setting
Patient engagement and follow through with case management	3	Barrier	V. Process
Challenges locating and contacting clients	3	Barrier	V. Process
Follow-up care after client inpatient care or appointments with healthcare system	3	Facilitator/Barrier	II. Outer Setting
Technology and informatics support; coordination with IT	3	Facilitator/Barrier	III. Inner Setting
Comprehensive system to identify all individuals who need help	2	Facilitator	I. Intervention Characteristics
Resources to support basic needs	2	Facilitator/Barrier	II. Outer Setting
Well-trained community members to refer individuals not being captured in the system	2	Facilitator	II. Outer Setting
Regular and sustainable funding	2	Facilitator/Barrier	I. Intervention Characteristics
Tribal and political leadership	2	Facilitator/Barrier	V. Process
Relationships with providers	2	Facilitator	III. Inner Setting
Data management and tracking	2	Facilitator	III. Inner Setting
Tribal resolution or mandate	2	Facilitator/Barrier	II. Outer Setting
Safety concerns during home visits	2	Barrier	V. Process
Sharing statistics across agencies and looking at data together to observe patterns	2	Barrier	II. Outer Setting
Lack of space and time or agreement on priorities	2	Barrier	III. Inner Setting
Sensitivity to diverse sociocultural norms	2	Facilitator/Barrier	II. Outer Setting

## Data Availability

The datasets generated during the current study are not publicly available due to tribal sovereignty and ownership of the data by the participating tribes. Supplementary Material Table S1 provides the complete list of factors prioritized by at least one tribal partner. Detailed data from individual focus groups will not be publicly available.

## Supplementary Materials

The following supporting information can be downloaded at https://www.mdpi.com/article/10.3390/ijerph21121616/s1: Table S1: Complete list of facilitators and barriers to implementing Celebrating Life; Figure S1: Steps towards adaptation and implementation of Celebrating Life.
