# Supplementary material for: Facilitators and Barriers to Implementing a Community Suicide Database and Prevention Program in Diverse Tribal Communities
Source: Int J Environ Res Public Health. 2024 Dec 3;21(12):1616. doi: 10.3390/ijerph21121616 (PMC11675467; doi:10.3390/ijerph21121616)
Supplement: Supplementary file 1 [file ijerph-21-01616-s001.zip › ijerph-3237649 Supplementary Material/supplementary materials table S1.pdf]

Table S1

Complete List of Facilitators and Barriers to Implementing Celebrating Life			
**Prioritized Factor**	**Number of Sites Endorsing**	**Barrier or Facilitator**	**CFIR Domain**
Dedicated and motivated staff	5	Facilitator	IV. Characteristics of Individuals
Coordination and regular meetings with other departments (MOUs & protocols), collaboration across diverse stakeholders	5	Facilitator/Barrier	II. Outer Setting
Availability and responsiveness of case managers, and turnaround between identification and contact	4	Facilitator	V. Process
Community awareness of program	4	Facilitator/Barrier	II. Outer Setting
Enough staff members to provide detailed case management and balance different work needs	3	Facilitator/Barrier	III. Inner Setting
Community trust of the program	3	Facilitator	II. Outer Setting
Clear policies and procedures for a variety of situations	3	Facilitator	III. Inner Setting
Training of case managers	3	Facilitator	III. Inner Setting
Community resistance and stigma related to suicide	3	Barrier	II. Outer Setting
Patient engagement and follow through with case management	3	Barrier	V. Process
Challenges locating and contacting clients	3	Barrier	V. Process
Follow-up care after client inpatient care or appointments with healthcare system	3	Facilitator/Barrier	II. Outer Setting
Technology and informatics support, coordination with IT	3	Facilitator/Barrier	III. Inner Setting
Comprehensive system to identify all individuals who need help	2	Facilitator	I. Intervention Characteristics
Resources to support basic needs	2	Facilitator/Barrier	II. Outer Setting
Well-trained community members to refer individuals not being captured in the system	2	Facilitator	II. Outer Setting
Regular and sustainable funding	2	Facilitator/Barrier	I. Intervention Characteristics
Tribal and political leadership	2	Facilitator/Barrier	V. Process
Relationships with providers	2	Facilitator	III. Inner Setting
Data management and tracking	2	Facilitator	III. Inner Setting
Tribal resolution or mandate	2	Facilitator/Barrier	II. Outer Setting
Safety concerns during home visits	2	Barrier	V. Process
Sharing statistics across agencies and looking at data together to observe patterns	2	Barrier	II. Outer Setting
Lack of space and time or agreement on priorities	2	Barrier	III. Inner Setting
Sensitivity to diverse socio-cultural norms	2	Facilitator/Barrier	II. Outer Setting

Departments implementing screening procedures to support reporting suicide behaviors to CL	1	Facilitator	II. Outer Setting
Identifying needs and highest risk population when implementing programs	1	Facilitator	V. Process
Community outreach	1	Facilitator	II. Outer Setting
Follow-up on patients at home and in schools	1	Facilitator	V. Process
Case managers' knowledge of community	1	Facilitator	IV. Characteristics of Individuals
Individual case work	1	Facilitator	V. Process
Serve the community	1	Facilitator	II. Outer Setting
Availability of clinical support	1	Facilitator	II. Outer setting
Consistency in daily work	1	Facilitator	V. Process
Making connections to services	1	Facilitator	II. Outer setting
Red tape is difficult to change quickly within the system (e.g. approvals, EHR system, stigma)	1	Barrier	III. Inner Setting
Family resistance	1	Barrier	V. Process
Parental consent	1	Barrier	V. Process
Communication with CIH support in Baltimore	1	Barrier	III. Inner Setting
Stress & burnout	1	Barrier	IV. Characteristics of Individuals
Dealing with death & spiritual burden	1	Barrier	IV. Characteristics of Individuals

Figure S1

Steps towards adaptation and implementation of Celebrating Life

Initial meetings

- Where is the community starting from? What is currently happening in the community around suicide prevention?
- How much of a concern is suicide in the community?
- How involved is leadership in addressing this concern?
- What is this team looking for in a suicide prevention program?

Building community buy-in

- Identify stakeholders in other agencies and organizations who are interesting in collaborating on this work
- Be able to communicate the initial goals for implementing this suicide prevention program in the community
- Hold community meetings and discussions to encourage conversations about suicide prevention efforts

Building the community's program

- Establish who will house and staff this program
- Understand how this program collaborate with other suicide prevention work in the community
- Secure funding for ongoing staffing and program efforts
- Finalize and seek approval for any resolutions or policies needed to support the program
- Complete trainings with Celebrating Life team until case managers are prepared to complete in-person follow-ups

Ongoing program support

- Continued virtual meetings (generally monthly) for any questions the team encounters
- Continuously check in with case management staff and support wellbeing
- Can assist with quarterly or annual reporting to leadership
