# Supplementary figures and images for: Facilitators and Barriers to Implementing a Community Suicide Database and Prevention Program in Diverse Tribal Communities
Source: Int J Environ Res Public Health. 2024 Dec 3;21(12):1616. doi: 10.3390/ijerph21121616 (PMC11675467; doi:10.3390/ijerph21121616)

**Figure S1**

Steps towards adaptation and implementation of Celebrating Life

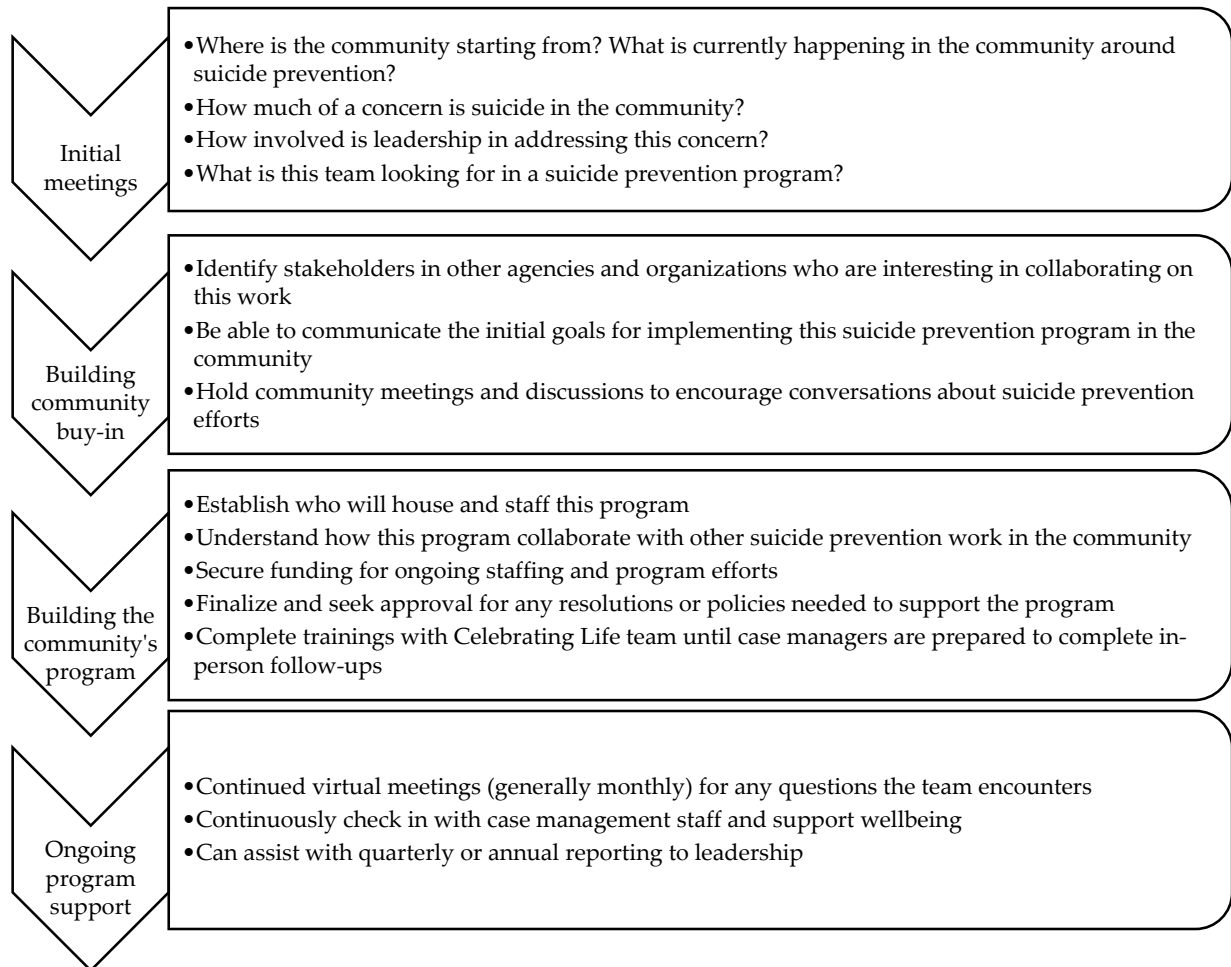

Supplement: Supplementary file 1 [file ijerph-21-01616-s001.zip › ijerph-3237649 Supplementary Material/Figure S1.pdf]
